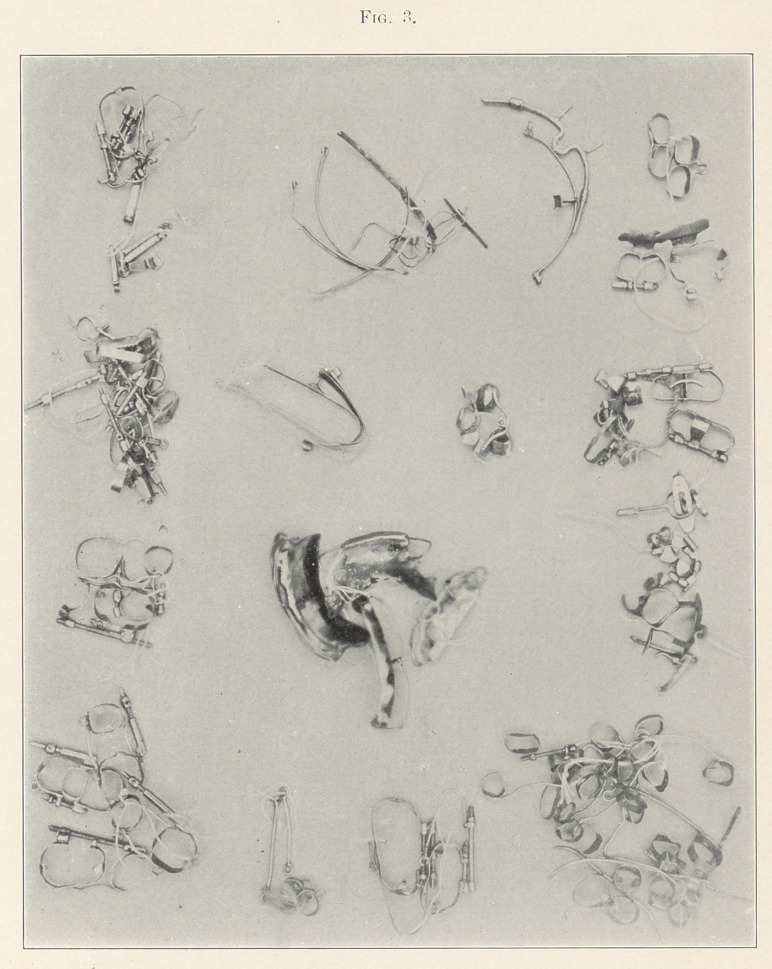# Dr. J. N. Farrar’s Appliances

**Published:** 1905-05

**Authors:** 


### DR. J. N. FARRAR'S APPLIANCES.

BY THE EDITOR.

There has frequently been a doubt expressed by some dentists, and even some specialists in orthodontia, that the great variety of appliances illustrated by this author are not really practical, or, in other words, had not been made use of in actual cases. In order to settle this question the writer had the pleasure of examining a large collection of these mechanisms, probably several hundred, with names and dates attached, constituting a remarkable exhibit not only in number, but in ingenuity in preparation. The value of this mass, which by no means includes all his mechanisms, lies not only in the fact that all are of 18-carat gold, but have decided interest in showing the very great development he has made in this special line of work. All of Dr. Farrar's mechanisms show great scientific merit in accuracy and ease of action.

It was thought that an exhibit of this kind would not only be instructive, but of interest to all skilful workers in this specialty, as well as settling all disputed questions as to the fact of their great practical use as regulating appliances at rest. Every one here represented has been used and proved by most successful results, in easy management and least discomfort to patient. The exhibit is unique in that it is probably the first that was ever offered to any dental periodical, similarily illustrated.

The frontispiece represents a tangled mass taken from Dr. Farrar's regulating drawers without any attempt at orderly arrangement, but still sufficiently indicative of their character and the use to which they had been applied. The photographs from which these illustrations were made are the first this author has ever presented in his various publications, as he has preferred to illustrate, by "working line plans," all articles in his papers and in his volumes, written upon Orthodontia, which are familiar to all readers of dental literature. His idea is that photography is not equal, as a clear guide, to line drawings for the use of students in mechanics. The common opinion is entertained that photographs represent nothing but the truth, but it is recognized by artists that the effect of photography by varied posings and "foreshortenings," and by "pencilling the negative" in illustrating machinery, architecture, and the human face, can bring about as numerous changes and untruthful results as the padding and coloring of actors upon the

FIG. 1.

Fig. 2.

Fig. 3.

theatrical stage can effect. The modern illustrations often seen in periodicals bear out this view. But the "half-tones," while recognized as by no means perfect, have served, and will doubtless continue to serve, as an aid in the illustration of practical papers, as photographs and line drawings of houses are mutual helps in architecture.

## Figures and Tables

**Fig. 1. f1:**
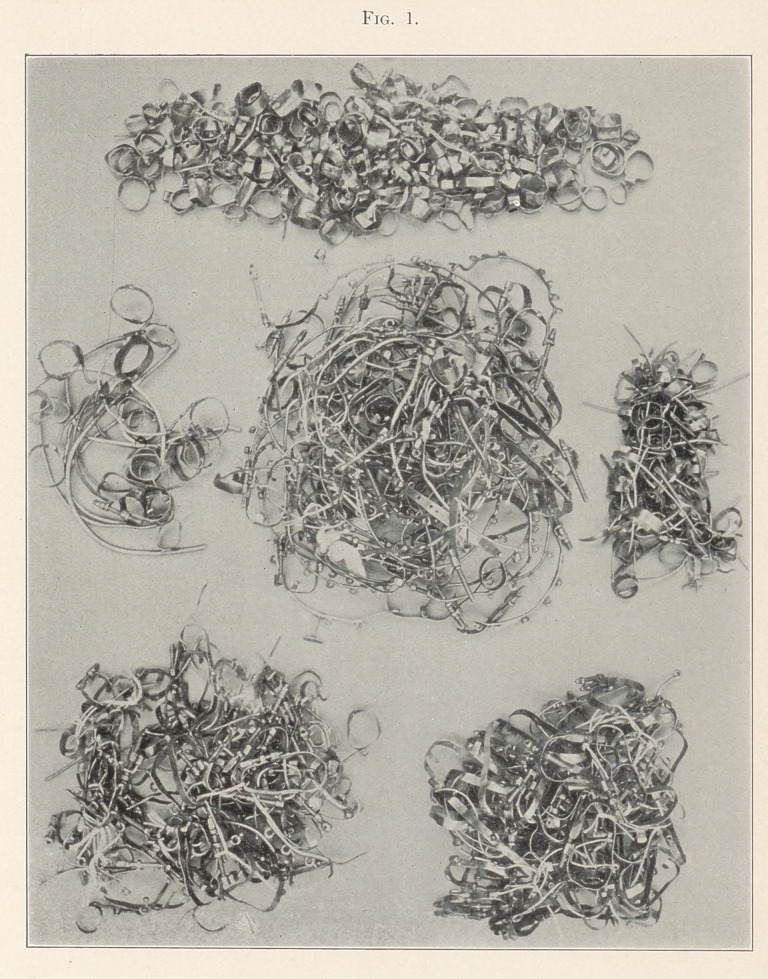


**Fig. 2. f2:**
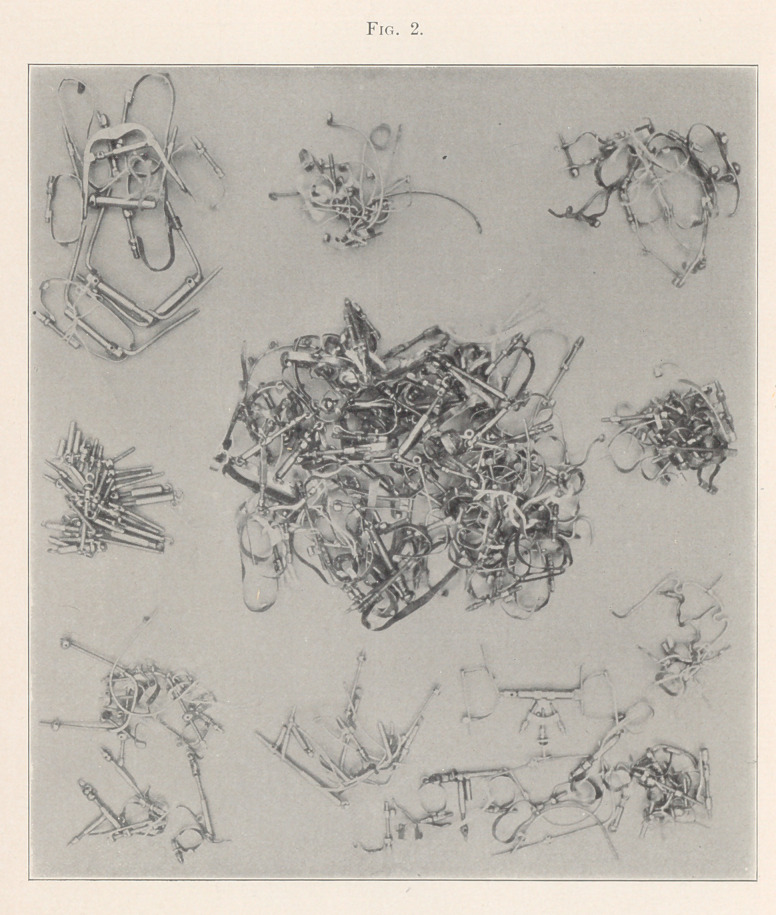


**Fig. 3. f3:**